# Family Health among Families with Primary School Children during the COVID Pandemic in Thailand, 2022

**DOI:** 10.3390/ijerph192215001

**Published:** 2022-11-15

**Authors:** Nareerut Pudpong, Sataporn Julchoo, Pigunkaew Sinam, Sonvanee Uansri, Watinee Kunpeuk, Rapeepong Suphanchaimat

**Affiliations:** 1 Sirindron College of Public Health, Chonburi 20000, Thailand; 2 International Health Policy Program, Ministry of Public Health, Nonthaburi 11000, Thailand; 3 Department of Disease Control, Division of Epidemiology, Nonthaburi 11000, Thailand

**Keywords:** family health, family well-being, primary school, children, parents, Thailand

## Abstract

Despite evidence suggesting that the COVID pandemic has negatively affected the mental health and well-being of school aged children and parents, there are limited studies describing the state of family well-being. This study aimed to use the family health lens to assess the well-being of Thai families with primary school children and to identify its associated factors. A cross-sectional survey was conducted during January and March 2022, a period of school closure when onsite education was replaced by online education from time to time. The family health scale (FHS) questionnaire survey was carried out among 701 parents of Thai families with primary school children. The questionnaire comprised 10 questions regarding family belief, health, relationships, financial security, and housing environment. Independent variables included: (1) parental/household factors; (2) online learning related issues; (3) children’s mental health; and (4) parents’ health behaviors. Multinomial logistic regression was undertaken. Results showed that half of Thai families (54.6%) reported having moderate health status. Factors that were associated with lower levels of family health, such as poor or moderate levels, included families with a child that had mental health problems (adjusted odd ratio (AOR) = 5.0 [95% CI = 2.6–9.5] for poor v. excellent, and AOR = 2.7 [95% CI = 1.9–4.0] for moderate v. excellent), single parents (AOR = 2.5 [95% CI = 1.2–5.2] for poor v. excellent), a higher number (≥3) of children (AOR = 2.1 [95% CI = 1.0–4.0] for moderate v. excellent), and smoking parents (AOR = 6.5 [95% CI =1.2–34.8] for poor v. excellent). During health emergencies, health policy for providing adequate assistance to single parents, especially those that have a child with mental health problems, is of utmost importance. The design of health promotion activities and interventions should be targeted not only at single families, but also families with higher numbers of children and parents who smoke at home.

## 1. Introduction

Between the first COVID outbreak in Wuhan, China in 2020 and now, there have been disruptions in many areas of human life, such as health, the economy, environment, and education [1,2,3]. Public health measures aimed at controlling the spread of the disease and reducing infected cases and deaths have also caused negative impacts on people’s health and well-being globally. Evidence suggests that social isolation due to travel restrictions, social distancing, and school closures has produced negative impacts on the mental health of both school children and their parents [4,5]. Compared to children in other age groups, primary school children have been particularly vulnerable during the pandemic, as school closures have led to several changes in their physical and social environments, which consequently affects their mental health. For instance, previous literature has shown that primary school children have been affected by public health restriction measures in various ways, including physical activities, sleeping patterns, eating habits, and psychological responses, as well as mental health issues, which include difficulty in concentrating, boredom, irritability, restlessness, nervousness, feelings of loneliness, uneasiness, and worries [6,7,8,9,10]. In addition, depression, anxiety, and stress have been reported among parents who take care of their school-aged children [5].

Despite there being a significant amount of evidence on the mental health of school children and their parents during the COVID pandemic, there are only a limited number of investigations into family health. Family health has a broader definition than just the physical and/or mental health of each individual member; it focuses on the collective quality of life of all members in the family. Karakas F. et al. (2004) suggested that family health and/or well-being can be viewed from three perspectives: effective parenting, love and being together, and peace and harmony [11]. Zuna N. et al. (2010) elaborated that family health refers to a “sense of well-being of the family, collectively and subjectively defined and informed by its members, in which individual and family-level needs interact” (p. 262) [12]. McGregor SLT. (2020) gathered several concepts of family health and proposed eight dimensions to define “family health”: (1) financial security and stability; (2) relational well-being (intra and interpersonal); (3) group dynamics and cohesion; (4) family autonomy; (5) collective, overall health; (6) community connection and belonging; (7) spiritual health; and (8) ecological well-being. However, assessing family health would largely depend on which concept the research team chose to use, which dimensions to assess, and/or which assessment tool to apply [13].

During the COVID pandemic, Gadermann AC. et al. (2022) examined the mental health dimension of family members through the viewpoint of parents in Canada, and found that families with children aged younger than 18 years old at home experienced deteriorating mental health, leading to an increase in alcohol consumption and suicidal thoughts among those parents [14]. Rizzo R. et al. (2021) conducted a self-assessment survey among parents/caregivers from a wider perspective on family health in Italy and reported some selective dimensions such as worrying about the stability of living situations (26.3%), a reduction in quality time spent with their child (7.1%), difficulty in balancing between childcare and work responsibilities (40.0%), and an increase in family financial problems (48.5%) [15]. Chen YC. et al. (2021) explored the COVID impacts on families with school-aged children in the US, and revealed that low-income and lower-middle class parents, and parents of color experienced more instrumental and financial difficulties as a result of job loss and/or the reduction of household incomes than white parents and those with higher income [16]. Pailhé A. et al. (2021) examined the situation of families with primary school children during the lockdown period in France, and reported that the reduction of family incomes negatively affected family relationship as well as the children’s social development [17]. In addition, Gayatri M. and Irawaty DK. (2021) conducted a literature review regarding family resilience during the pandemic and suggested that family well-being should be built up through good and healthy communication, and participation in positive activities together among family members; this would lead to a sense of togetherness, trust, cohesion, and happiness [18].

To the best of our knowledge, there have been very few studies in Thailand exploring family health during the COVID pandemic. Ruksee N. et al. (2021) examined family relationships in addition to the stress and anxiety of family members during the first lockdown period of the pandemic, and revealed that 56.2% of Thai families reported having stress at a mild level, followed by 29.9% at a moderate level, and 13.9% at a predominant level. In addition, when compared with the pre-lockdown period, care among family members increased (58.4%), while inter-family quarrels remained the same (25.2%) or lessened (21.1%). It was also found that factors associated with their stress included age, occupation, number of children in the house, and anxiety [19].

Since research that describes holistic family well-being either in Thailand and elsewhere is still lacking, particularly during the COVID pandemic, this study aims to use family health lens to assess the well-being of Thai families with primary school children during the COVID pandemic and identify its associated factors. Findings from this study would help increase more understandings in the field of ‘family health’ globally, especially during the emergency situation. Furthermore, the findings will be useful for informing policy and for designing appropriate public interventions to provide adequate assistance for Thai families to overcome undesirable health emergencies that may possibly occur again in the future.

## 2. Methods

### 2.1. Study Design and Participants

We conducted a cross-sectional survey between January and March 2022. Parents and/or guardians with primary school children at home were recruited from ten participating schools in five major provinces in Thailand. In each province, one public school and one private school were selected based on the suggestion of local healthcare providers or local education officers and the willingness of school directors to participate in the research. The five studied provinces were located in different regions of the country and comprised Bangkok (Central), Chiang Rai (North), Udon Thani (Northeast), Chonburi (East), and Songkhla (South).

Sample size was calculated using the following formula: *n* = z^2^*p* (1 − *p*)/d^2^; where ‘z’= 1.96 (reflecting the z-statistic for two-tailed 95% confidence interval level), ‘*p*’ reflects the prevalence of family health in Thailand, and ‘d’ denotes acceptable error. As previously mentioned, the Thai study by Ruksee N. et al. (2021) during the first lockdown of the COVID pandemic showed that the prevalence of Thai families reported having stress at a predominant level of 13.9%. Therefore, we replaced ‘*p*’ with 0.139, and also replaced ‘d’ with 0.04. After applying all parameters in the formula, a sample size of 288 was needed. However, after accounting for a 20% non-response rate and incomplete information, the final sample size was expanded to 350. We then employed simple random sampling with probability proportional to size (PPS) (based on the number of children in participating schools) for selecting parents from schools’ name lists.

Initially, we planned to distribute paper-based questionnaires to all participating parents. However, due to the fluctuation of infected cases as well as the changes of restriction measures enforced by the Government in response to the severity of the COVID situation at that time, we could not physically visit some schools. We then changed the research plan by distributing online questionnaires via Google Forms instead. Consequently, we were able to acquire 730 participants, which was far larger than the calculated sample size. To ensure data quality when answering the questionnaires, we adopted the instructional manipulation check (IMC) method by inserting one question to ask the participants “not to answer the question and leave it blank”. Therefore, we discarded 27 questionnaires where the parents still answered this IMC question. We also discarded two more questionnaires due to eligibility, since those parents reported not having any primary school children at home. Hence, at the end, there were a total of 701 samples in this study (paper based = 231; online = 470). The summary of participants by school location (Table S1a) and type of questionnaire (Table S1b) can be seen in Supplementary Table S1.

### 2.2. Data Collection

Data collection began with the research team explaining the survey methods to the designated teacher at all participating schools. As previously mentioned, the paper-based questionnaires were distributed to parents for schools that we could physically visit, while the link for Google Forms or the online questionnaire was sent to the designated teacher at the schools that were contacted remotely. The designated teacher took responsibility for randomly distributing the survey questionnaires to all parents; the paper-based questionnaires were provided to parents via face-to-face meeting, and the online questionnaires were sent to parents via their regular communication platform (e.g., email or LINE application). Parents were given a maximum of two weeks to complete the questionnaire. The designated teachers then collected the paper-based questionaries and turned them over to the research team, whereas online questionnaires were directly received by the research team through the Google Forms platform.

Data collection was carried out after we received ethical approval from the Institute of the Development of Human Research Protections (IHRP), Thailand (letter head—IHRP 1045/2564). All participating parents were informed about the purpose and risks as well as the benefits of the study by the designated teacher. Written consent and the information sheet were distributed to all participants together with the paper-based questionnaires. Parents who answered the paper-based questionnaires received a stipend of approximately USD 8 for their time. For online questionnaires, the information sheet was provided electronically and appeared before the parents began answering the survey questions. If the parents proceeded to the following webpage after reading the online information sheet, this indicated that they provided consent.

### 2.3. Measurements

We adopted the Family Health Scale (FHS) questionnaire where its short form was proposed by Crandall et al. (2020) [20]. This tool aims to measure ‘family health’ in a more holistic way by considering four factors: (1) family social and emotional health processes; (2) family healthy lifestyle; (3) family health resources; and (4) family external social supports [20]. The questionnaire asked the children’s parents or guardians about their family situation during the pandemic (see in Supplementary File S2). The FHS questionnaire comprised 10 questions relating to family beliefs, health, relationships, financial security, and housing environment. Parents were asked to rate each question in five-point Likert scales (1 for strongly disagree, 5 for strongly agree). We translated the questionnaire into the Thai language and had three experts review the validity of its content. Subsequently, the questionnaire was piloted among 30 parents of primary school children in a Thai province of Thailand that was not included in this study. A reliability test of this set of questions was also undertaken, and resulted in a Cronbach’s alpha coefficient of 0.75.

The FHS cutoffs were determined by first applying reverse coding to three negative questions (Questions 6, 9, 10), and then coding “1” for questions with a rating score of 4 or 5 and “0” for those with a rating score less than 4. Then, family health was measured by taking the sum (maximum of 10 points) and then divided into three groups: (i) poor (0–5 points); (ii) moderate (6–8 points); and (iii) excellent (9–10 points).

The main groups of independent variables in this study consisted of: (1) parental/household factors; (2) online learning related issues; (3) children’s mental health; and (4) parent’s health behaviors. Parental/household factors included gender, age (20–34, 35–44, and ≥45 years), education (never/primary school, high school/diploma, and bachelor or higher), monthly incomes (≤10,000, 10,001–30,000, and ≥30,001 Baht), parental status (with partner/single), family type (single/extended), and household size by number of people in the house (1–3, 4–5, and >5 people). Online learning-related factors included number of digital devices in the house, e.g., mobile phones/computers (0–1 and ≥2 devices), frequency of assistance provided to the child during the learning time by parents (every day/not every day), and whether or not parents assisted the child by themselves (yes/no). Children’s mental health was measured by the parents’ reports based on the youngest child with mental health problems, as measured by the Strengths and Difficulties Questionnaire (SDQ) [21] (yes/no). Parent’s health behaviors comprised smoking (yes/no) and alcohol drinking (yes/no).

### 2.4. Data Analysis

Descriptive statistics (number and frequencies) were used to describe the characteristics of parents and their household, online learning related issues, children’s mental health, and parents’ health behaviors. The prevalence of family health among Thai families was calculated. Chi-square test and univariable multinomial logistic regression was employed to explore the association between family health groups across each independent variable. The variables that showed statistical significance (*p*-value < 0.05) in the univariable analysis would be included in the multivariable analysis by multinomial logistic regression to account for the effect of the independent variables all at once. We used the ‘excellent’ group as the reference for multinomial logistic regression. Crude odds ratios (COR), adjusted odds ratio (AOR) and 95% confidence interval (95% CI) were reported. All analyses were performed using STATA version 13.1 (license number: 401406358220).

## 3. Results

### 3.1. Characteristics of Parents, Household, Online Related Issues, and Children

Table 1 presents the characteristics of the parents, their household and children, and online learning-related issues. The majority of participating parents were female (80.2%) and of working age (54.4%; 35–44 years). Most of them (61.2%) had completed their bachelor’s degree. Almost half of the parents (49.5%) had monthly incomes of about THB 10,000–30,000 (USD 265–794), while approximately one-third of them (30.4%) received over THB 30,000/month. Most parents (79.0%) raised their children with partners. In terms of family type, slightly over half of the participants were from extended families (54.8%), while approximately half of the participants had four or five family members (50.9%). Single families constituted about 43.5% of the participants. Regarding online learning-related factors, approximately half of the parents (50.2%) reported having at most one electronic device at home. Most of the parents (89.0%) reported assisting their children with learning at home by themselves, and doing so every day (81.5%). In addition, about 41.1% of participants had children with mental health problems. Only a small proportion of parents reported that they smoked (3.0%), and less than one-fifth drank alcohol (17.8%).

### 3.2. Parent-Reported Family Health

The prevalence of levels of family health is shown in Table 2. More than half of the parents considered their family health to be at a moderate level during the COVID pandemic (54.6%), followed by the excellent (35.4%) and poor (9.1%) levels, respectively.

### 3.3. Factors Associated with Family Health

The crude analysis of family health groups across all of the characteristics studied in this study are presented in Table 3. Parental education (*p*-value < 0.05), income (*p*-value < 0.05), and relationship status with partner (*p*-value < 0.001) were significantly related to family health. Regarding online learning factors, only the number of electronic devices at home exhibited a significant association with family health (*p*-value < 0.05). The youngest child having mental health problems was significantly associated with family health (*p*-value < 0.001). In addition, smoking among parents appeared to be associated with family health (*p*-value < 0.001), but not drinking of alcohol.

After adjusting for all studied variables, variations in family health across parental education and incomes were reduced to non-significant levels (see Table 4). However, parental status in taking care of the children remained significant, as family health among those taking care of the children on their own was likely to be poor (COR = 3.4 [95% CI = 1.9–6.1]; AOR = 2.5 [95% CI = 1.2–5.2]) compared to those taking care of the children with their partners. The number of school-aged children at home also exhibited a significant association with family health during the pandemic. Compared to families with one or two children at home, Thai families with three or more school-aged children were found to be in moderate health, with COR of 1.9 [95% CI = 1.1–3.2], and AOR of 2.1 [95% CI = 1.0–4.0]. Both the crude and adjusted models showed that the health of families where the youngest child had mental health problems had greater odds of being poor (COR = 4.8 [95% CI = 2.7–8.6]; AOR = 5.0 [95% CI = 2.6–9.5]) or moderate (COR = 2.5 [95% CI = 1.8–3.6]; AOR = 2.7 [95% CI = 1.9–4.0]. In terms of parental health behavior, parents who smoked seemed to report having ‘poor’ family health in both the crude model (COR = 5.2 [95% CI = 1.3–19.8]) and adjusted model (AOR = 6.5 [95% CI = 1.2–34.8]).

## 4. Discussion

This study appears to be among the first studies, not only in Thailand but in Asia, to attempt to examine ‘family health’ as a whole instead of looking only at the health of an individual. In the midst of the COVID pandemic, the study found that the health of Thai families with primary school children was likely to be at a moderate level (54.6%). Moreover, it also found that the level of family health was more likely to be viewed as poor or moderate and exhibited an association with certain factors such as being a single parent, more numbers of school-aged children (≥3) at home, having a child with mental health problems in the family, and parenting with unhealthy behaviors like smoking.

In this study, having a child with mental health problems was found to have a strong association with poor or moderate family health. This may be explained by one important domain of the ‘family health’ concept: the overall, collective health of each individual in the family [12,13]. The imperfect health condition of a family member, either physically or mentally, could have a negative influence on the health of the family as a whole. Furthermore, it is possible that the positive atmosphere or activities at home could also be ruined by the emotional or behavioral actions of a child with mental health in the family. A qualitative study by Wäsche H. et al. (2021) also revealed that family health climate could be shaped by both individual health related-interactions and environmental factors [22]. This present study suggests that the assessment and monitoring of the health condition of all family members or the population before, during, and post-pandemic, is of critical public health importance. Additionally, health promotion activities or interventions to support healthy individuals and their families would also benefit the nation since the build-up of quality human capital starts from the family as it is the smallest unit in society.

Parts of our findings are relatively well in line with an Italian study, which revealed that parents faced difficulty in balancing their care and work responsibilities (40.0%) [15] if they were unable to seek support from their partners. This situation is likely to worsen among families containing large numbers of school-aged children at home and among single parents, where the search for the balance between maintaining a job to obtain sufficient family income and taking care of children while coping with social restrictions and promoting home-based learning during the pandemic is extremely difficult. Hence policies and/or public health interventions focusing on a provision of adequate assistance to single parents should be urgently exercised, especially during health emergency situations. Furthermore, public health interventions should target not only single families but also families with large numbers of school-aged children. This is because during school closures, children must spend most of their time at home, and parents are generally required to play a significant role in assisting their children with learning.

While previous studies have suggested that financial hardships during the pandemic appeared to be a significant issue in relation to family health in terms of creating negative impacts on family relationships and mental health for both parents and children [16,17], this study found a significant association between family health and income only in the crude analysis. This could possibly be due to the differences in the methodological approach. Previous studies investigated only selective aspects of family health such as the mental health of individual members or relationships among them, whereas our study summed the scores of the self-rated overview of family health, including their relationships with outsiders, healthcare seeking behavior, financial security, and sufficient housing space. Thus, additional research on family health and the financial aspect would be invaluable.

With regard to parental health behavior, family health in this study seemed to be related to the smoking behavior of parents, but not alcohol drinking. However, it is important to be noted that the parental smoking (3%) found in this study is relatively low compared to that found in the general Thai population (19.1%) during normal period [23]. In addition, this discovery differed from a Canadian study conducted during the pandemic, which suggested a significant association between alcohol consumption among parents and poor family health [14]. However, that study did not focus on parental smoking as primary objective. This inconsistency between the findings may once again be due to the difference in methodological approaches, such as the difference in family health domains of interest as well as the difference of measurement tools; it could also be possibly related to the difference in culture and the way of life of each country’s population.

There are some limitations to this study. First, since the study took place in Thailand during the pandemic, the public health implications based on the results might vary due to the difference in settings. Second, we included only children’s mental health and parental health behaviors in the analysis but did not include the physical health of both children and parents. As mentioned above, the health condition of each individual would likely affect ‘family health’, and future research should consider including all individual health conditions in the analysis. Third, this study is subject to some degree of social desirability bias as the information is derived from the parents’ views, which might exhibit some bias in favor of the investigator’s expectations. Fourth, the generalizability of the study is also limited by the fact that there are various concepts of ‘family health’ and the tools used for measuring it also varied from study to study. For example, although one Thai study conducted during the first lockdown found that Thai families experienced stress at mild (56.2%), moderate (29.9%), and predominant levels of (13.9%), the questions used for assessing ‘stress’ might not be the same as the ones used to assess ‘health’ in this study [19]. Apart from using different questions in the questionnaires, another Thai study conducted during the normal period also classified family health as moderate, good, and excellent [24]; this meant that the method used for data analysis would have also varied. Hence, more research in this area is warranted. Additionally, since ‘family health’ as well as its related issues are relatively complex, future research studies that employ a mix-method design or collect qualitative information would be very helpful for further discussion in this field.

## 5. Conclusions

The state of family health among Thai families with primary school children during the COVID pandemic in 2022 was at a moderate level. This may potentially be due to the difficulties experienced by all individuals in adjusting their lives to the new normal lifestyle with regards to the public health restrictions enacted during the period. Factors affecting family health included being a single parent, a higher number of school-aged children (≥3) at home, having a child with mental health problems in the family, and parents with unhealthy behavior like smoking. The results suggest that during health emergency situations, public health policy that promotes adequate assistance to single parents should focus on families that have a child with mental health problems in addition to families with single parents. Additionally, the design of health promotion activities and interventions should target families with large numbers of children and smoking parents. More research to monitor family well-being as a whole should be continuously undertaken to detect changes over time as well as to identify associated factors in order to provide appropriate intervention to help families in a timely manner.

## Figures and Tables

**Table 1:** Characteristics of parents, household, children, and online learning related issues (*n* = 701).

Characteristics	*n*	%
***Parental/Household***		
**Gender**		
Female	562	80.2
Male	139	19.8
**Age (year)**		
20–34	120	17.1
35–44	381	54.4
45 and over	184	26.3
Not answer	16	2.3
**Education**		
Never attended/Primary school	41	5.9
High school/diploma	231	33.0
Bachelor and higher	429	61.2
Incomes/month (THB *)		
≤10,000	139	19.8
10,001–30,000	347	49.5
≥30,001	213	30.4
Not answer	2	0.3
**Parental status**		
With partner	554	79.0
Single	146	20.8
Not answer	1	0.1
**Family in the house**		
Single family	305	43.5
Extended family	384	54.8
Not answer	12	1.7
**Household size (people)**		
1–3	146	20.8
4–5	357	50.9
>5	185	26.4
Not answer	13	1.9
***Online learning related issues***		
**Mobiles/Computers in the house**		
0–1 device	352	50.2
2 devices or more	349	49.8
**Number of all school children in the house**		
1 child	276	39.4
2 children	320	45.7
3 children or more	94	13.4
Not answer	11	1.6
**Assist children in online learning**		
Everyday	571	81.5
Not everyday	118	16.8
Not answer	12	1.7
**Assist children by myself**		
Yes	624	89.0
No	76	10.8
Not answer	1	0.1
***Children’s mental health***		
**Having the youngest child with mental health problems measured by Strengths and difficulties questionnaire (SDQ)**		
Normal	406	57.9
At risk/have problems	288	41.1
Not answer	7	1.0
***Parents’ health behaviors***		
**Smoking**		
No	679	96.9
Yes	21	3.0
Not answer	1	0.1
**Alcohol drinking**		
No	576	82.2
Yes	125	17.8

* USD 1 = THB 37.44.

**Table 2:** Parent-reported family health among Thai families with primary school children during the COVID pandemic in 2022.

Family Health	*n*	%
Poor	64	9.1
Moderate	383	54.6
Excellent	248	35.4
Not answer	6	0.9

**Table 3:** Family health across the studied characteristics (*n* = 701).

Characteristics	Poor (%)	Moderate (%)	Excellent (%)	*p* Value ^b^
***Parental/Household***				
**Gender**				0.221
Female	51 (79.7)	298 (77.8)	207 (83.5)	
Male	13 (20.3)	85 (22.2)	41 (16.5)	
**Age (year)**				0.133
20–34	16 (25.4)	70 (18.7)	32 (13.3)	
35–44	31 (49.2)	212 (56.5)	137 (56.9)	
45 and over	16 (25.4)	93 (24.8)	72 (29.8)	
**Education**				0.005
Never attended/Primary school	5 (7.8)	24 (6.3)	12 (4.8)	
High school/diploma	26 (40.6)	141 (36.8)	61 (24.6)	
Bachelor and higer	33 (51.6)	218 (56.9)	175 (70.6)	
**Incomes/month (THB ^a^)**				0.001
≤10,000	18 (28.1)	84 (21.9)	36 (14.5)	
10,001–30,000	34 (53.1)	196 (51.2)	115 (46.4)	
>30,000	12 (18.8)	103 (26.9)	97 (39.1)	
**Parental status**				0.000
With partner	37 (57.8)	310 (81.2)	204 (82.3)	
Single	27 (42.2)	72 (18.8)	44 (17.7)	
**Family in the house**				0.378
Single family	25 (39.7)	176 (46.7)	102 (42.0)	
Extended family	38 (60.3)	201 (53.3)	141 (58.0)	
**Household size (people)**				0.972
1–3	15 (23.8)	78 (20.7)	52 (21.4)	
4–5	33 (52.4)	196 (52.0)	125 (51.4)	
>5	15 (23.8)	103 (27.3)	66 (27.2)	
***Online learning related issues***				
**Mobiles/Computers in the house**				0.003
0–1 device	43 (67.2)	195 (50.9)	109 (43.9)	
2 devices or more	21 (32.8)	188 (49.1)	139 (56.1)	
**Number of all school children**				0.186
1 child	25 (39.0)	140 (37.5)	108 (43.7)	
2 children	28 (43.8)	174 (46.7)	115 (46.6)	
3 children or more	11 (17.2)	59 (15.8)	24 (9.7)	
**Assist children in online learning**				0.060
Everyday	45 (72.6)	314 (83.1)	208 (85.2)	
Not everyday	17 (27.4)	64 (16.9)	36 (14.8)	
**Assist children by myself**				0.472
Yes	57 (89.1)	337 (88.0)	225 (91.1)	
No	7 (10.9)	46 (12.0)	22 (8.9)	
***Children’s mental health***				
**Having the youngest child with mental health problems measured by Strengths and difficulties questionnaire (SDQ)**				<0.001
Normal	23 (36.5)	196 (51.8)	181 (73.3)	
At risk/have problems	40 (63.5)	182 (48.2)	66 (26.7)	
***Parent’s health behavior***				
**Smoking**				0.035
No	59 (92.2)	370 (96.9)	244 (98.4)	
Yes	5 (7.8)	12 (3.1)	4 (1.6)	
**Alcohol drinking**				0.404
No	50 (78.1)	321 (83.8)	200 (80.7)	
Yes	14 (21.9)	62 (16.2)	48 (19.3)	

^a^ USD 1 = THB 37.44, ^b^ Compared using Pearson’s chi-squared test.

**Table 4:** Multinomial logistic regression on family health among Thai families with primary school children during the COVID pandemic in 2022.

Group	Variable	Poor vs. Excellent				Moderate vs. Excellent			
		Crude OR	*p* Value	Adjusted OR	*p* Value	Crude OR	*p* Value	Adjusted OR	*p* Value
**Parental/Household factors**	**Gender**								
	Female	1.0				1.0			
	Male	1.3 [0.6–2.6]	0.477			1.4 [1.0–2.2]	0.083		
	**Age (year)**								
	20–34	1.0				1.0			
	35–44	0.5 [0.2–0.9]	0.030	0.6 [0.3–1.4]	0.217	0.7 [0.4–1.1]	0.149	0.8 [0.5–1.3]	0.326
	45 and over	0.4 [0.2–1.0]	0.049	0.5 [0.2–1.4]	0.198	0.6 [0.4–1.0]	0.047	0.6 [0.3–1.1]	0.082
	**Education**								
	Never/Primary school	1.0				1.0			
	High school/Diploma	1.0 [0.3–3.2]	0.969			1.2 [0.5–2.5]	0.707		
	Bachelor or higher	0.5 [0.1–1.4]	0.161			0.6 [0.3–1.3]	0.198		
	Incomes (THB *)								
	≤10,000	1.0				1.0			
	10,001–30,000	0.6 [0.3–1.2]	0.132	0.8 [0.3–1.9]	0.663	0.7 [0.5–1.1]	0.174	0.7 [0.4–1.3]	0.280
	>30,000	0.2 [0.1–0.6]	0.001	0.5 [0.2–1.5]	0.337	0.5 [0.3–0.7]	0.001	0.5 [0.3–1.0]	0.055
	**Parental status**								
	With partner	1.0				1.0			
	Alone	3.4 [1.9–6.1]	<0.001	2.5 [1.2–5.2]	0.017	1.1 [0.7–1.6]	0.726		
	**Family type**								
	Single	1.0				1.0			
	Extended	1.1 [0.6–1.9]	0.742			0.8 [0.6–1.1]	0.250		
	**Household size**								
	1–3 people	1.0				1.0			
	4–5 people	0.9 [0.5–1.8]	0.801			1.0 [0.7–1.6]	0.835		
	>5 people	0.8 [0.4–1.8]	0.560			1.0 [0.7–1.7]	0.868		
**Online learning related factors**	**Mobiles/Computers in the house**								
	0–1 device	1.0				1.0			
	2 devices or more	0.4 [0.2–0.7]	0.001	0.6 [0.3–1.1]	0.093	0.8 [0.5–1.0]	0.088		
	**Number of school age children**								
	1 child	1.0				1.0			
	2 children	1.1 [0.6–1.9]	0.869			1.2 [0.8–1.6]	0.379	1.3 [0.8–1.0]	0.270
	3 children or more	2.0 [0.9–4.6]	0.109			1.9 [1.1–3.2]	0.019	2.1 [1.0–4.0]	0.036
	**Assist children in online learning**								
	Everyday	1.0				1.0			
	Not everyday	2.2 [1.1–4.2]	0.021		0.063	1.2 [0.8–1.8]	0.471		
	**Assist children by myself**								
	Yes	1.0				1.0			
	No	1.3 [0.5–3.1]	0.619			1.4 [0.8–2.4]	0.222		
**Having a child with mental health problem**	**Reported the youngest child with mental health problems**								
	Normal	1.0							
	At risk/have problems	4.8 [2.7–8.6]	<0.001	5.0 [2.6–9.5]	<0.001	2.5 [1.8–3.6]	<0.001	2.7 [1.9–4.0]	<0.001
**Parents health behaviors**	**Smoking**								
	No	1.0				1.0			
	Yes	5.2 [1.3–19.8]	0.017	6.5 [1.2–34.8]	0.027	2.0 [0.6–6.2]	0.242		
	**Alcohol drinking**								
	No	1.0				1.0			
	Yes	1.2 [0.6–2.3]	0.653			0.8 [0.5–1.2]	0.306		

* USD 1 = THB 37.44.

## Data Availability

Data available on request due to ethical restrictions.

## Supplementary Materials

The following supporting information can be downloaded at: https://www.mdpi.com/article/10.3390/ijerph192215001/s1, Supplementary File S1: Participants by school type, location, and questionnaire types; Supplementary File S2: Family Health Scale—Short-Form (FHS-SH).
