# Supplementary material for: Family Health among Families with Primary School Children during the COVID Pandemic in Thailand, 2022
Source: Int J Environ Res Public Health. 2022 Nov 15;19(22):15001. doi: 10.3390/ijerph192215001 (PMC9690226; doi:10.3390/ijerph192215001)
Supplement: Supplementary file 1 [file ijerph-19-15001-s001.zip › ijerph-2004804-supplementary.pdf]

Supplementary file

Supplementary S1: Participants by school type, location, and questionnaire types

Table S1a: Percentages of participants by school type and location

Province	School		n	%
	Public	Private		
Bangkok	12	43	55	7.8
Chiang Rai	50	45	95	13.5
Udon Thani	229	138	367	52.4
Chonburi	48	43	91	13.0
Songkhla	50	43	93	13.3
**Total**	**389**	**312**	**701**	**100.0**

Table S1b: Percentages of participants in the survey by school location and type of questionnaires

Province	Type of questionaries				รวม
	paper-based (n)	%	online (n)	%	
Bangkok	43	78.2	12	21.8	55
Chiang Rai	95	100.0	0	0.0	95
Udon Thani	0	0.0	367	100.0	367
Chonburi	0	0.0	91	100.0	91
Songkhla	93	100.0	0	0.0	93
**Total**	**231**	**32.9**	**470**	**67.1**	**701**

__Supplementary S2:__ **Family Health Scale – Short-Form (FHS-SH)**

	**Strongly Disagree (1)**	**Somewhat Disagree (2)**	**Neither Agree nor Disagree (3)**	**Somewhat Agree (4)**	**Strongly Agree (5)**
**In my family…**					
1. We support each other.					
2. I feel safe in my family relationships.					
3. We help each other in seeking healthcare service when needed (such as making doctor's appointment).					
4. We help each other make healthy changes.					
5. We stay hopeful even in difficult times.					
6. We do __not__ trust doctors and other health professionals					
7. We have people outside of our family that we can turn to when we have problems at school or work.					
8. If we need financial help, we have people outside of our family that we could turn to for a loan (e.g., for $200).					
**In the past 12 months....**					
9. My family did __not__ have enough money at the end of the month after bills were paid.					
10. My family did __not__ have adequate housing.					
